# Genomic and transcriptomic analysis reveal molecular basis of salinity tolerance in a novel strong salt-tolerant rice landrace Changmaogu

**DOI:** 10.1186/s12284-019-0360-4

**Published:** 2019-12-27

**Authors:** Bing-Rui Sun, Chong-Yun Fu, Zhi-Lan Fan, Yu Chen, Wen-Feng Chen, Jing Zhang, Li-Qun Jiang, Shuwei Lv, Da-Jian Pan, Chen Li

**Affiliations:** 1 Rice Research Institute, Guangdong Academy of Agricultural Sciences, Guangzhou, 510640 China; 2 Guangdong Provincial Key Laboratory of New Technology in Rice Breeding, Guangzhou, China

**Keywords:** Rice landrace, Salt tolerance, BSA-seq, Transcriptome sequencing

## Abstract

**Background:**

Salt stress is an important factor that limits rice yield. We identified a novel, strongly salt tolerant rice landrace called Changmaogu (CMG) collected from a coastal beach of Zhanjiang, Guangdong Province, China. The salt tolerance of CMG was much better than that of the international recognized salt tolerant rice cultivar Pokkali in the germination and seedling stages.

**Results:**

To understand the molecular basis of salt tolerance in CMG, we performed BSA-seq for two extreme bulks derived from the cross between CMG and a cultivar sensitive to salt, Zhefu802. Transcriptomic sequencing was conducted for CMG at the germination and young seedling stages. Six candidate regions for salt tolerance were mapped on Chromosome 1 by BSA-seq using the extreme populations. Based on the polymorphisms identified between both parents, we detected 32 genes containing nonsynonymous coding single nucleotide polymorphisms (SNPs) and frameshift mutations in the open reading frame (ORF) regions. With transcriptomic sequencing, we detected a large number of differentially expressed genes (DEGs) at the germination and seedling stages under salt stress. KEGG analysis indicated two of 69 DEGs shared at the germination and seedling stages were significantly enriched in the pathway of carotenoid biosynthesis. Of the 169 overlapping DEGs among three sample points at the seedling stage, 13 and six DEGs were clustered into the pathways of ABA signal transduction and carotenoid biosynthesis, respectively. Of the 32 genes carrying sequence variation, only *OsPP2C8* (Os01g0656200) was differentially expressed in the young seedling stage under salt stress and also showed sequence polymorphism in the ORFs between CMG and Zhefu802.

**Conclusion:**

*OsPP2C8* was identified as the target candidate gene for salinity tolerance in the seedling stage. This provides an important genetic resource for the breeding of novel salt tolerant rice cultivars.

Salinity is one of the most common abiotic stresses limiting crop production. Except for in coastal areas, improper irrigation and the use of poor-quality water aggravate the salinization of arid and semi-arid soil in inland areas (Rahman et al., 2016). Rice (*Oryza sativa* L.) is a staple food crop that is salt sensitive in both young seedling and reproductive stages (Kumar et al., 2013). Identifying rice germplasm that is salt tolerant and breeding rice cultivars that are salt tolerant are the most economic and effective methods for the reduction of rice yield loss caused by salinity.

Rice salinity tolerance is a complex trait controlled by quantitative trait loci (QTLs) (Roy et al., 2011; Wang et al., 2013) and also shows different physiological mechanisms (Li and Xu, 2007). Although many salt tolerant QTLs have been detected in different rice lines (Koyama et al., 2001; Ammar et al., 2009; Pandit et al., 2010; Gong et al., 1999; Kumar et al., 2015), only several major salt tolerant rice QTLs or genes such as *qSKC1* (Lin et al., 2004; Ren et al., 2005), *qSNC7* (Lin et al., 2004), *Saltol* (Thomson et al., 2010), and *OsRR22* (Takagi et al., 2015) have been identified by genomic methods. A major QTL, *qSKC1*, encodes a sodium transporter in rice under salt stress (Ren et al., 2005). *OsRR22* encodes a B-type response regulator protein that acts as a transcription factor regulating genes involved in osmotic responses and/or ion transport between parenchyma cells and vascular tissue cells of roots (Takagi et al., 2015).

Next generation sequencing (NGS) technologies contribute to discovering genome-wide genetic variation and genotyping in a highly efficient way (Huang and Han, 2013). The relatively low cost of sequencing enables the use of genome and transcriptomic sequencing to map some agronomic traits, especially quantitative traits (Varshney et al., 2014b; Pandey et al., 2017). The bulk segregant analysis (BSA) method is more effective in rapidly locating candidate genomic regions that underlie the target genes based on whole genomic resequencing for the extreme bulks and both parents (Takagi et al., 2013). Several transcriptomic studies have identified numerous differentially expressed genes in salinity-tolerant rice varieties compared to salinity-sensitive rice varieties (Kawasaki et al., 2001; Walia et al., 2005; Cotsaftis et al., 2011; Wang et al., 2016; Shankar et al., 2016). These differentially expressed genes (DEGs) are generally associated with stress signaling, ion transport, transcription regulation, and some specific metabolic processes (Kawasaki et al., 2001; Walia et al., 2005; Cotsaftis et al., 2011; Kumar et al., 2013). However, current studies of the tolerance of rice to salinity mainly focus on the classic salt-tolerant rice cultivar, Pokkali, and its derived line, FL478. We identified a new salt-tolerant landrace called Changmaogu (CMG), which shows much stronger tolerance to salinity than Pokkali at the germination and young seedling stages (Fig. 1a).

**Fig. 1:**
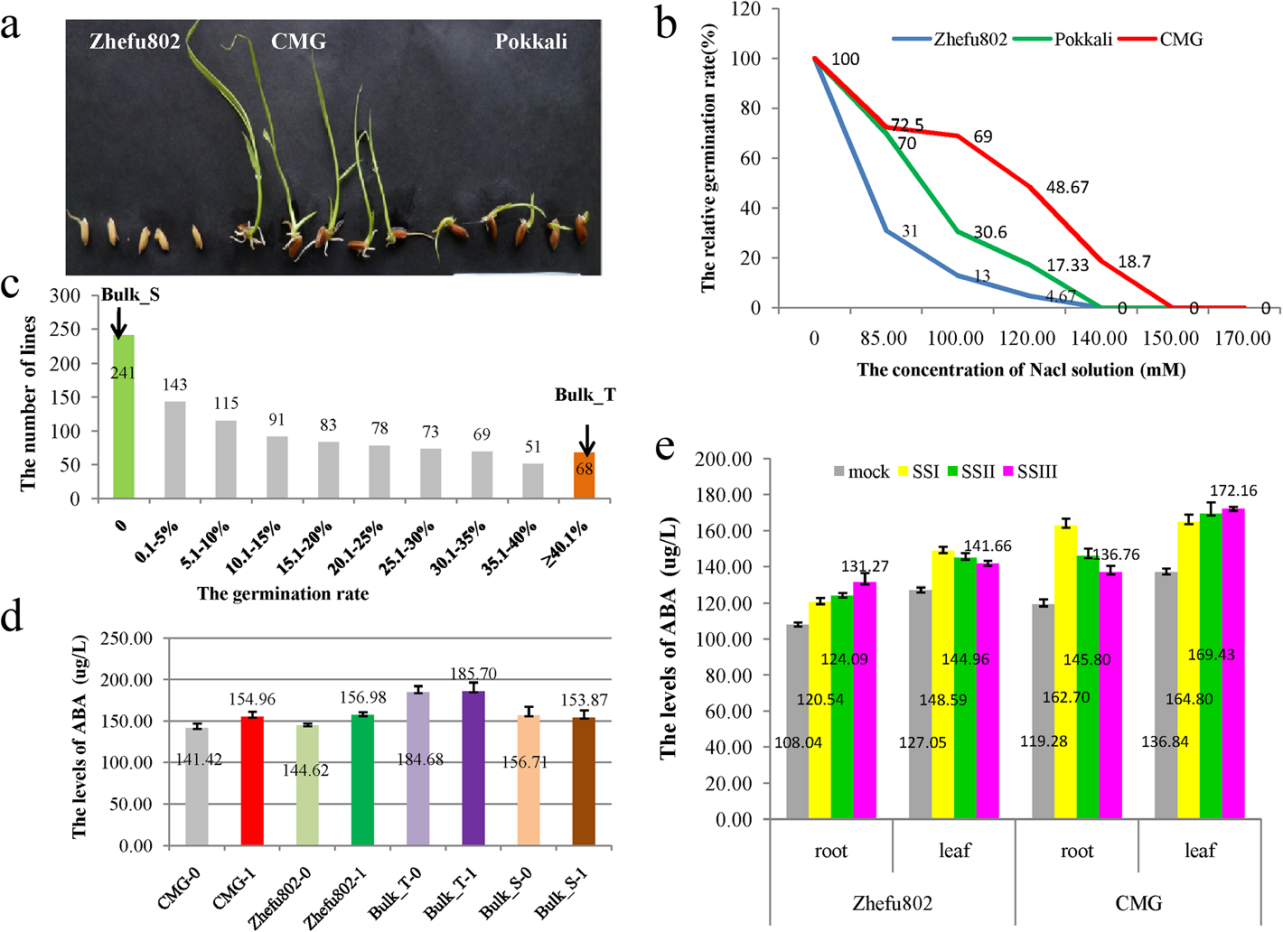
The identification of salt tolerance and the assay of abscisic acid (ABA) in Zhefu802 and Changmaogu (CMG). **a**: The seedlings of CMG, Zhefu802, and Pokkali when the dry seeds were immersed in sea water (total salt:2%) at room temperature for 30 days; **b**: The germination tendencies of CMG, Zhefu802, and Pokkali at different NaCl concentrations; **c**: The germination rates of CMG, Zhefu802, and Pokkali in 0.12 mol/L NaCl solution; **d**: the ABA levels of both parents and two bulks at germination stage; e: the ABA levels in roots and leaves of CMG at different sampling points under salt stress

In this study, we performed BSA sequencing for two extreme bulks and transcriptomic sequencing for CMG in the germination and young seedling stages under salt stress. This research aimed to identify the salt tolerant candidate genes in the seedling stage and provide insight into the molecular basis of salinity tolerance in CMG.

## Results

### Evaluation of Salinity Tolerance of CMG and Construction of the Extreme Mapping Population for BSA-Seq

The rice landrace Changmaogu (CMG), collected from a coastal beach of Zhanjiang, Guangdong Province, China, showed strong tolerance to the salinity of sea water (the total salt concentration: 370 mM) (Fig. 1a). To investigate the salt tolerance of CMG and identify the optimal salt concentration, the germination rates of CMG, Pokkali, and Zhefu802 were scored under six different salt (NaCl) concentrations (85 mM, 100 mM, 120 mM, 140 mM, 150 mM, and 170 mM). The germination rates of the three rice lines gradually decreased with increasing salt concentration. Overall, CMG showed a much stronger tolerance to salinity than Pokkali and Zhefu802 under all six salt concentrations (Fig. 1b). The germination rate of CMG was similar to that of Pokkali and much greater than that of Zhefu802 in the 85 mM NaCl treatment. In the 100 mM NaCl treatment, the germination rate of CMG was higher than that of Pokkali. The germination rate of CMG varied based on the NaCl concentration; the germination rates were 69%, 48.67%, and 18.7% in the 100, 120, and 140 mM NaCl treatments, respectively. Hence, the 120 mM NaCl treatment was considered the optimal concentration. The germination rate of CMG, Pokkali, and Zhefu802 were 48.67%, 17.33%, and 4.67%, respectively, in the 120 mM NaCl solution (Fig. 1b).

To map the genes related to salinity tolerance in CMG, we constructed an F_2_ population derived from the cross between the strongly tolerant cultivar CMG and the sensitive cultivar Zhefu802. This F_2_ population was used to identify the degree of salinity tolerance in the young seedling stage using F_2:3_ seeds in the 120 mM NaCl treatment. We identified the salinity tolerance of more than 1000 F_2:3_ seeds and detected 241 and 68 F_2:3_ lines showing no germination and more than 40% germination rate, respectively. We continued to cultivate the 68 F_2:3_ seeds for 25 days after salt treatment and selected 30 extremely tolerant individuals showing good growth for 25 days after salt stress and 30 individuals sensitive to salinity to construct the tolerant and sensitive salinity bulks for BSA-seq (Fig. 1c).

### Measurement of Abscisic Acid (ABA)

Abscisic acid (ABA) is a plant hormone that regulates plant growth and development and is rapidly increased by abiotic stresses such as drought and salinity (Mahajan and Tuteja, 2005). High salinity increases ABA (Kumar et al., 2013). To investigate whether the salt tolerance of CMG is associated with ABA, we tested the content of ABA in the roots and leaves under both normal growth conditions and salt stress at the germination and young seedling stage in CMG and Zhefu802. At the germination stage, the ABA levels in CMG and Zhefu802 increased under salt stress, compares with normal growth condition. But the ABA levels in CMG were equivalent to those in Zhefu802 under salt stress and normal growth condition, respectively. Compared with under normal growth condition, the ABA levels in both extreme bulks did not significantly change under salt stress. But the ABA levels (about 185 μg/L) in extreme salt tolerant bulk were remarkably higher than those (about 154 μg/L) in salt sensitive bulk under salt stress and normal growth condition (Fig. 1d). At the young seedling stage, compared with normal growth conditions, the ABA levels in roots and leaves increased under salt stress in both CMG and Zhefu802, and the increase of ABA in CMG was significantly higher than that in Zhefu802 (Fig. 1e). In roots of CMG, the ABA content instantly reached the highest level (162.70 μg/L) at the first sampling point (SSI) and then gradually reduced at SSII (145.80 μg/L) and SSIII (136.76 μg/L). In leaves of CMG, the ABA level gradually increased at three sampling points, SSI (164.80 μg/L), SSII (169.43 μg/L), and SSIII (172.16 μg/L).

However, Zhufu802 showed the different changing trend of ABA content in roots and leaves, compared with CMG. Namely, the ABA level gradually increased in roots (120.54 μg/L, 124.09 μg/L and 131.27 μg/L) and decreased in leaves (148.59 μg/L, 144.96 μg/L and 141.66 μg/L) at the sampling points from SSI to SSIII.

### Sequencing and Mapping of Reads to the ‘Nipponbare’ Reference Genome

Whole genome re-sequencing data were generated for both parents and the extreme salinity tolerant bulk (T_bulk) and salinity sensitive bulk (S_bulk) (Table 1). A total of 42.15 million clean reads were generated for the salinity tolerant parent, CMG, 35.79 million clean reads for the salt sensitive parent, Zhefu802, 71.88 million clean reads for S_bulk, and 72.86 million clean reads for T_bulk. The obtained sequencing data were 12.63 Gb for CMG, 10.72 Gb for Zhefu802, 21.54 Gbfor S_bulk, and 21.83Gb for T_bulk, and more than 96% clean reads were mapped to the Nipponbare reference genome. The rates of high quality (Q30) bases were more than 91% (91.82% for CMG, 93.66% for Zhefu802, 92.37% for S_bulk, and 92.78% for T_bulk).

**Table 1:** Coverage of the reads mapping to the Nipponbare reference genome from resequencing of the *indica* rice variety Zhefu802 and a rice landrace CMG using Hiseq 2000.

BMK ID	Clean_Reads	Clean_Base	Mapped (%)	Properly_mapped (%)	Q30(%)
CMG	42,143,655	12,625,063,756	96.39	90.33	91.82
Zhefu802	35,794,170	10,720,972,860	97.42	91.65	93.66
S-pool	71,884,976	21,536,286,334	98.01	91.94	92.37
R-pool	72,863,530	21,826,349,910	97.71	92.03	92.78

### Candidate Regions for Salinity Tolerance by Bulked Segregant Analysis (BSA) Based on Genomic Resequencing

**Fig. 2:**
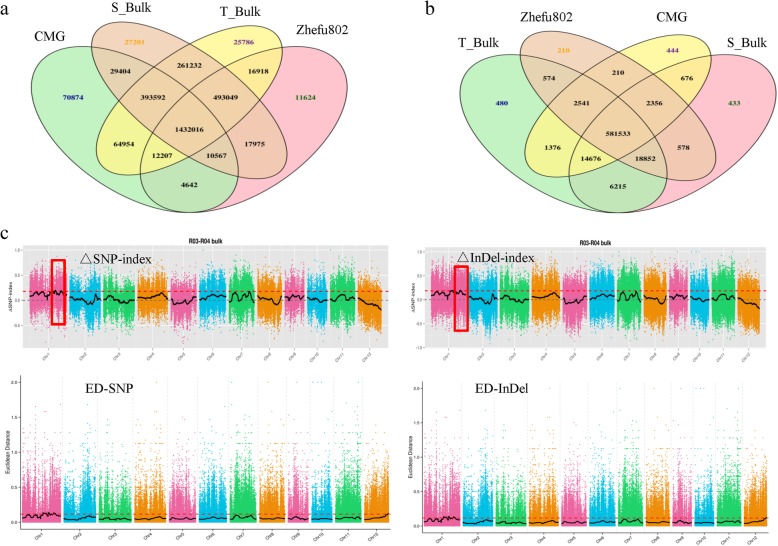
Constructing the extremely sensitive (S_bulk) and tolerant (T_bulk) salt bulks and BSA-seq analysis for both parents, CMG and Zhefu802, and the two extreme bulks. **a**: The frequency distribution of the germination rate in more than 1000 F_2:3_ lines from CMG and Zhefu802; **b**: Venn diagram of the number of SNPs in CMG, Zhefu802, S_bulk, and T_bulk; **c**: Venn diagram of the number of small InDels in CMG, Zhefu802, S_bulk, and T_bulk; **d**: the distribution of associated values based on SNP−/InDel-index and Euclidean Distance (ED) on different chromosomes

In total, we detected 1,043,738 SNPs, including 96,226 nonsynonymous coding SNPs and 253,340 small InDels, between both parent lines. Between the two extreme bulks, we detected 331,923 SNPs including 32,239 nonsynonymous coding SNPs and 82,091 small InDels (Tables 2 and 3; Fig. 2a,b). An association analysis of salinity tolerance and polymorphic markers was performed using Euclidean Distance (ED) and SNP/InDel-index methods. We detected seven and six related candidate regions covering 3.66 Mb and 10.16 Mb based on SNP-index and ED, respectively (Additional file 1: Table S1). Similarly, according to polymorphic InDels, we obtained 10 and nine candidate regions covering 3.63 Mb and 9.58 Mb based on InDel-index and ED, respectively (Additional file 1: Table S2). We overlapped the results based on the two methods, identifying six and six candidate regions covering 3.66 Mb and 2.6 Mb, respectively, based on the threshold value of the confidence interval of 0.8914 of Δ(SNP/InDel-index) at the 95% significant level (Fig. 2c;Additional file 1: Table S3). Finally, with further analyses, we identified that the six overlapped regions as the most likely candidate regions for salinity tolerance at the seedling stage. These six candidate regions were located on chromosome 1 and covered a 2.6 Mb region containing 430 genes (Table 4). Based on the sequence polymorphism in ORFs of 430 genes between CMG and Zhefu802, we found that 23 genes carried nonsynonymous coding SNPs and eight genes contained frame shift mutations (Table 5).

**Table 2:** The distribution of detected SNPs between both parents Zhefu802 and CMG and between the two extreme pools

Type	Zhefu802_vs_CMG	S-pool_vs_R-pool
Intergenic	57,105	17,740
Intragenic	36	8
Intron	168,873	51,854
Upstream	345,387	109,660
Downstream	268,158	84,689
UTR_5_Prime	7979	2607
UTR_3_Prime	17,650	5114
Splice_site_acceptor	563	165
Splice_site_donor	542	165
Splice_site_region	3889	1224
Start_gained	1809	551
Start_lost	254	101
Non_synonymous_start	17	5
Synonymous_coding	69,741	24,034
Non_synonymous_coding	96,226	32,239
Synonymous_stop	97	34
Stop_gained	5114	1603
Stop_lost	298	128
Other	0	2
Total	1,043,738	331,923

**Table 3:** The distribution of detected InDels between both parents Zhefu802 and CMG and between the two extreme bulks

Type	Zhefu802_vs_CMG	S-bulk_vs_R-bulk
Intergenic	14,431	4650
Intragenic	126	46
Intron	46,092	14,549
Upstream	92,827	30,240
Downstream	71,637	22,681
Utr_5_prime	5138	1906
Utr_3_prime	6685	2067
Splice_site_acceptor	123	42
Splice_site_donor	163	51
Splice_site_region	816	261
Start_lost	70	26
Frame_shift	8918	3219
Codon_deletion	1952	719
Codon_insertion	2147	829
Exon_deleted	1	0
Codon_change_plus_codon_insertion	636	251
Codon_change_plus_codon_deletion	1280	430
Stop_gained	224	86
Stop_lost	74	37
Other	0	1
Total	253,340	82,091

**Table 4:** The detailed positions of the candidate regions for salt tolerance in CMG

Chromosome	Start	End	Size (Mb)
Chr.1	26,110,000	26,130,000	0.020001
Chr.1	26,160,000	26,180,000	0.020001
Chr.1	26,490,000	27,300,000	0.810001
Chr.1	29,440,000	30,630,000	1.190001
Chr.1	35,800,000	35,940,000	0.140001
Chr.1	36,140,000	36,160,000	0.020001
Total	–	–	2.200006

**Table 5:** The candidate genes for salt tolerance based on the index and ED of SNP and InDel

Mutation type	Gene ID	Position	Fuctional annotation
nonsynonymous coding SNP	Os01g0655400	26,608,476–26,601,904	Transposon protein, putative
	Os01g0733200	30,582,485–30,583,743	HSF-type DNA-binding domain containing protein
	Os01g0656200	26,652,944–26,658,313	Protein phosphatase 2C, putative, expressed
	Os01g0655500	26,611,799–26,618,704	Serine/threonine-protein kinase stt7
	Os01g0647800	26,114,190–26,111,942	Hypothetical protein
	Os01g0655600	26,619,462–26,620,234	Hypothetical protein
	Os01g0729800	30,422,055–30,422,387	Hypothetical protein
	Os01g0658300	26,769,190–26,767,671	Microneme protein Sm70 putative, expressed
	Os01g0715100	30,516,831–30,517,430	Ubiquitin-related 4 (Precursor)
	Os01g0715100	29,740,123–29,742,527	Conserved hypothetical protein
	Os01g0733100	30,585,423–30,585,100	Hypothetical protein
	Os01g0655700	26,622,829–26,624,411	Hypothetical protein
	Os01g0656600	26,706,158–26,699,189	Hypothetical protein
	Os01g0665500	27,200,990–27,196,417	Probable WRKY transcription factor 71
	Os01g0714700	29,717,779–29,718,346	Hypothetical protein
	Os01g0659900	26,872,217–26,875,033	F-box domain and kelch repeat containing protein
	Os01g0727600	30,341,594–30,340,015	Conserved hypothetical protein
	Os01g0712250	29,590,343–29,589,249	Arginine/serine-rich protein
	Os01g0722100	30,102,785–30,107,361	Bacterial transferase hexapeptide domain containing protein
	Os01g0732100	30,533,818–30,536,908	Hypothetical protein
	Os01g0655250	26,592,688–26,593,702	PWWP domain containing protein
	Os01g0655300	26,599,136-26,599,632	Similar to Trithorax 4
	Os01g0719150	29,971,297-29,971,882	Hypothetical protein
Frameshift mutation	Os01g0733200	30,582,485–30,583,743	HSF-type DNA-binding domain containing protein
	Os01g0659400	26,823,851–26,822,203	Non-protein coding transcript
	Os01g0655700	26,622,829–26,624,411	Hypothetical protein
	Os01g0655600	26,619,462–26,620,234	Hypothetical protein
	Os01g0733100	30,585,423–30,585,100	Cortical cell-delineating protein (Precursor)
	Os01g0712250	29,590,343–29,589,249	Arginine/serine-rich protein 45
	Os01g0655250	26,592,688–26,593,702	PWWP domain containing protein
	Os01g0655300	26,599,136-26,599,632	Similar to Trithorax 4

### Transcriptome Sequencing for CMG under Salt Stress at Germination and Seedling Stages

**Fig. 3:**
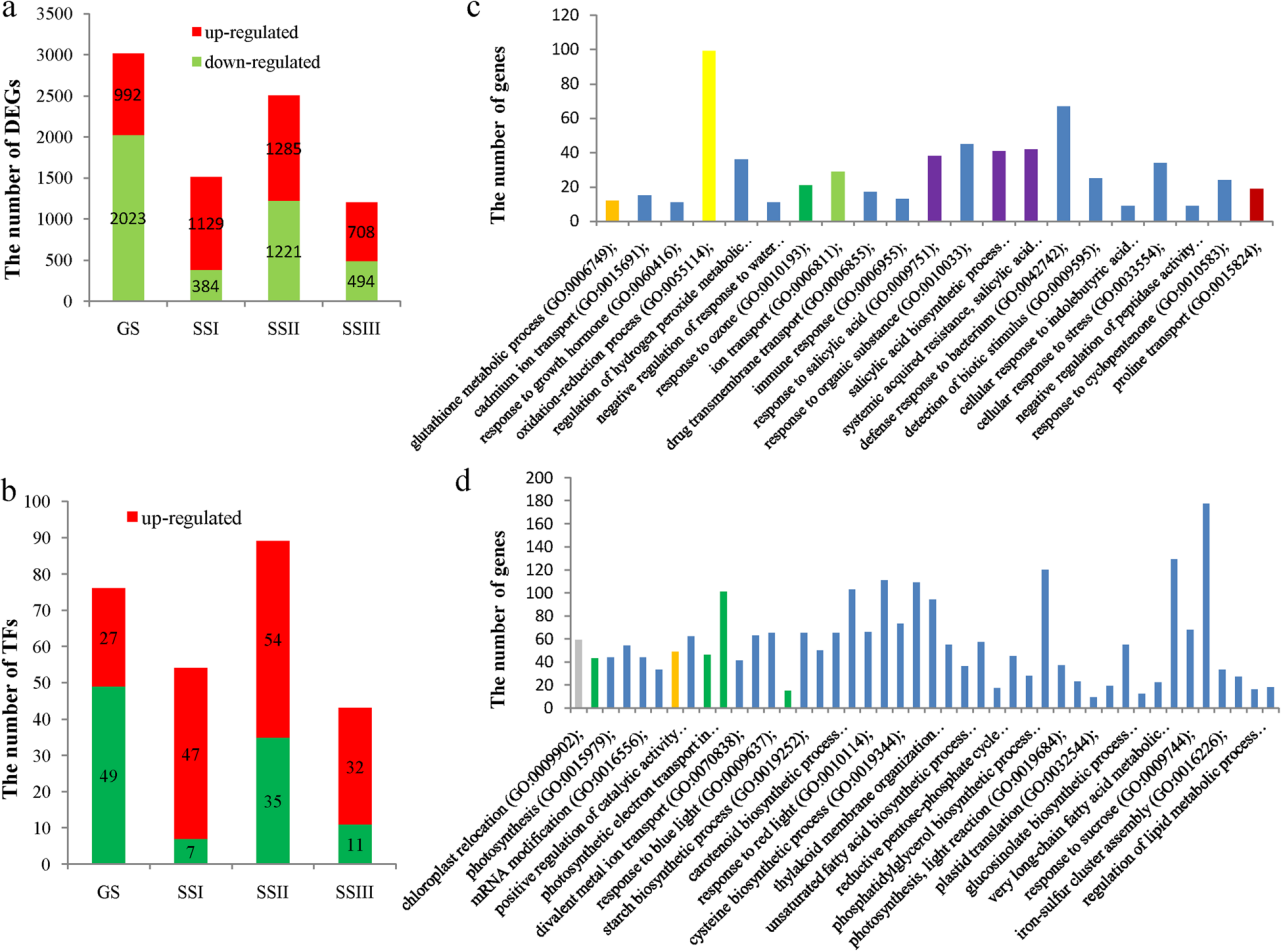
The number of differentially expressed genes (DEGs) at the germination and seedling stages and the GO analysis of DEGs at germination. **a**: The number of DEGs at the four sampling points; **b**: the number of differentially expressed transcription factors at the four sampling points; **c**: GO enrichment of upregulated DEGs at the germination stage; **d**: GO enrichment of downregulated DEGs at germination. Note: GS: germination stage; SSI: the sampling point I at seedling stage; SSII: the sampling point II at seedling stage; SSIII: the sampling point III at seedling stage

To understand the differentially expressed genes responding to salt stress, we performed transcriptomic sequencing under the conditions of normal growth and salt stress (120 mM NaCl solution). We randomly selected 24 DEGs for real time PCR (RT-PCR) to validate the differential expression (Additional file 1: Table S4). The RT-PCR results were in good agreement with the transcriptomic sequencing results (Table 6). We investigated the number of upexpressed and downexpressed genes at germination and the seedling stage and found that the number of downregulated genes was greater than that of upregulated genes at the germination stage. The number of upregulated genes was greater than that of downregulated genes at the seedling stage, especially at the first sampling point (SSI) (Fig. 3a). Similarly, the number of differentially expressed transcription factors (TFs) had a similar tendency at the germination and seedling stages (Fig. 3b).

**Table 6:** The expression value of 24 randomly selected genes in transcriptomic sequencing and RT-PCR

Gene ID	Transcriptomic sequencing (FPKM)				RT-PCR			
	Mock	SSI	SSII	SSIII	Mock	SSI	SSII	SSIII
Os01g0135700	8.68	21.53	26.66	29.28	1.00	3.04	3.58	3.87
Os01g0656200	2.21	6.82	8.56	4.98	1.00	2.86	3.21	2.33
Os01g0699400	2.13	20.09	20.49	25.22	1.00	8.97	8.69	9.54
Os01g0705700	0.15	0.98	1.67	4.13	1.00	4.31	6.74	9.24
Os01g0756300	8.88	16.91	34.55	18.67	1.00	2.13	5.57	4.31
Os01g0846300	16.04	54.68	135.19	72.51	1.00	2.98	7.64	7.52
Os02g0179600	1.65	3.71	7.33	14.93	1.00	2.16	3.68	5.96
Os02g0618400	4.77	21.75	6.26	10.57	1.00	3.86	2.31	2.67
Os02g0682300	27.73	111.55	43.08	53.33	1.00	4.25	1.98	2.69
Os02g0766700	21.46	44.27	138.76	52.40	1.00	2.23	4.39	2.28
Os03g0197100	3.50	6.48	34.52	10.20	1.00	1.98	5.68	2.54
Os03g0327800	22.15	65.80	51.06	39.94	1.00	3.57	2.11	4.65
Os04g0508500	1.92	8.16	48.37	5.11	1.00	5.64	10.85	8.65
Os04g0585050	15.28	50.67	14.57	22.81	1.00	2.38	1.64	1.36
Os05g0361700	10.28	21.08	85.17	25.56	1.00	2.26	5.39	4.57
Os05g0381400	1.30	11.28	59.61	44.86	1.00	6.31	42.68	38.05
Os05g0457200	0.21	1.04	10.28	7.35	1.00	2.98	15.37	6.92
Os06g0553100	2.62	13.43	23.85	8.42	1.00	3.36	8.65	5.65
Os09g0332300	31.55	67.81	32.45	22.78	1.00	2.11	1.02	0.76
Os09g0455300	1.59	4.85	19.77	4.57	1.00	2.38	5.78	5.64
Os01g0908600	11.46	4.93	1.55	6.53	1.00	0.55	0.12	0.74
Os03g0297600	78.65	24.70	11.37	31.74	1.00	0.32	0.36	0.44
Os10g0552800	9.99	3.85	3.06	4.48	1.00	0.31	0.26	0.22
Os01g0655500	14.26	17.89	22.52	27.83	1.00	1.59	1.64	1.91

A Gene Ontology (GO) analysis was performed to explore the biological processes related to salinity tolerance at germination and seedling stages. At germination stage, the DEGs were significantly enriched in 48 biological process terms (FDR < 0.001), including oxidation-reduction process (GO:0055114)(276 genes), carotenoid biosynthetic process (GO:0016117) (73 genes), glucosinolate biosynthetic process (GO:0019761) (71 genes), glutathione biosynthetic process (GO: 0006750) (71 DEGs), and response to karrikin (GO:0080167) (87 genes). The upregulated DEGs were significantly enriched into 21 terms (FDR < 0.001) (Fig. 3c), including glutathione metabolic process (GO:0006749), cadmium ion transport (GO:0015691), oxidation-reduction process (GO:0055114), negative regulation of response to water deprivation (GO:0080148), and proline transport (GO:0015824). The downregulated DEGs were clustered into 46 GO terms (FDR < 0.001) (Fig. 3d). Most of these enriched terms were associated with the processes of photosynthesis and catalytic activity.

**Fig. 4:**
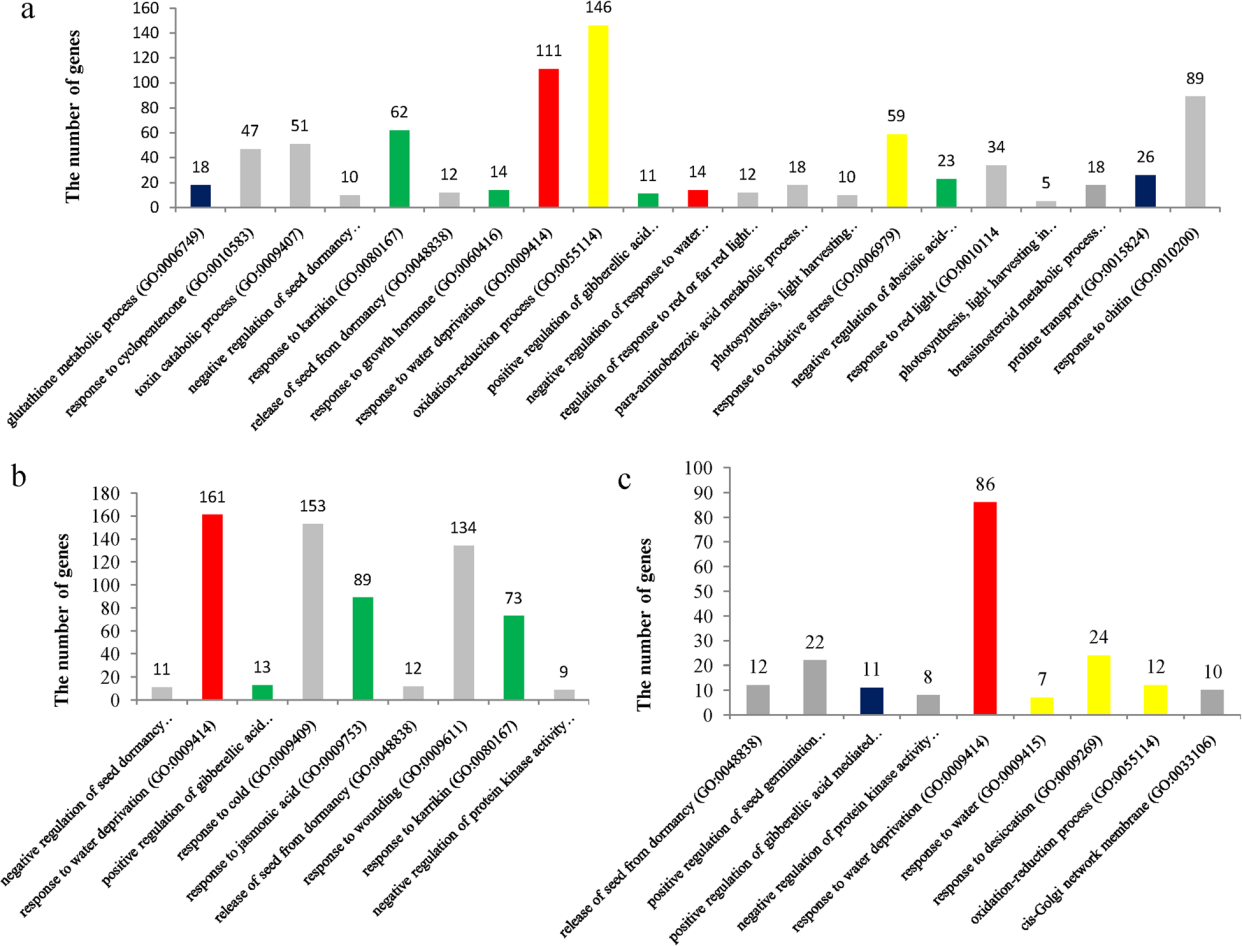
GO analysis of the detected DEGs at the seedling stage. **a**: GO enrichment of DEGs at the sampling point SSI; **b**: DEGs enriched in biological processes at the sampling point SSII; **c**: DEGs enriched in biological processes at the sampling point SSIII

At the first sampling point (SSI) in the seedling stage, the DEGs were grouped into 21 GO terms (FDR < 0.001) (Fig. 4a), including glutathione metabolic process (GO:0006749) (18 genes), response to karrikin (GO:0080167) (62 genes), response to water deprivation (GO:0009414) (111 genes), oxidation-reduction process (GO:0055114) (146 genes), negative regulation of response to water deprivation (GO:0080148) (14 genes), negative regulation of abscisic acid-activated signaling pathway (GO:0009788) (23 genes), and proline transport (GO:0015824) (26 genes). At SSII, these DEGs were clustered into nine GO terms (FDR < 0.001) containing response to water deprivation (GO:0009414) (161 genes), response to jasmonic acid (GO:0009753) (89 genes), and response to karrikin (GO:0080167) (73 genes) (Fig. 4b). At SSIII, the DEGs were also enriched into nine GO terms (FDR < 0.001) (Fig. 4c), including response to water deprivation (GO:0009414) (89 genes), response to water (GO:0009415) (seven genes), response to desiccation (GO:0009269) (24 genes) and oxidation-reduction process (GO:0055114) (112 genes).

**Fig. 5:**
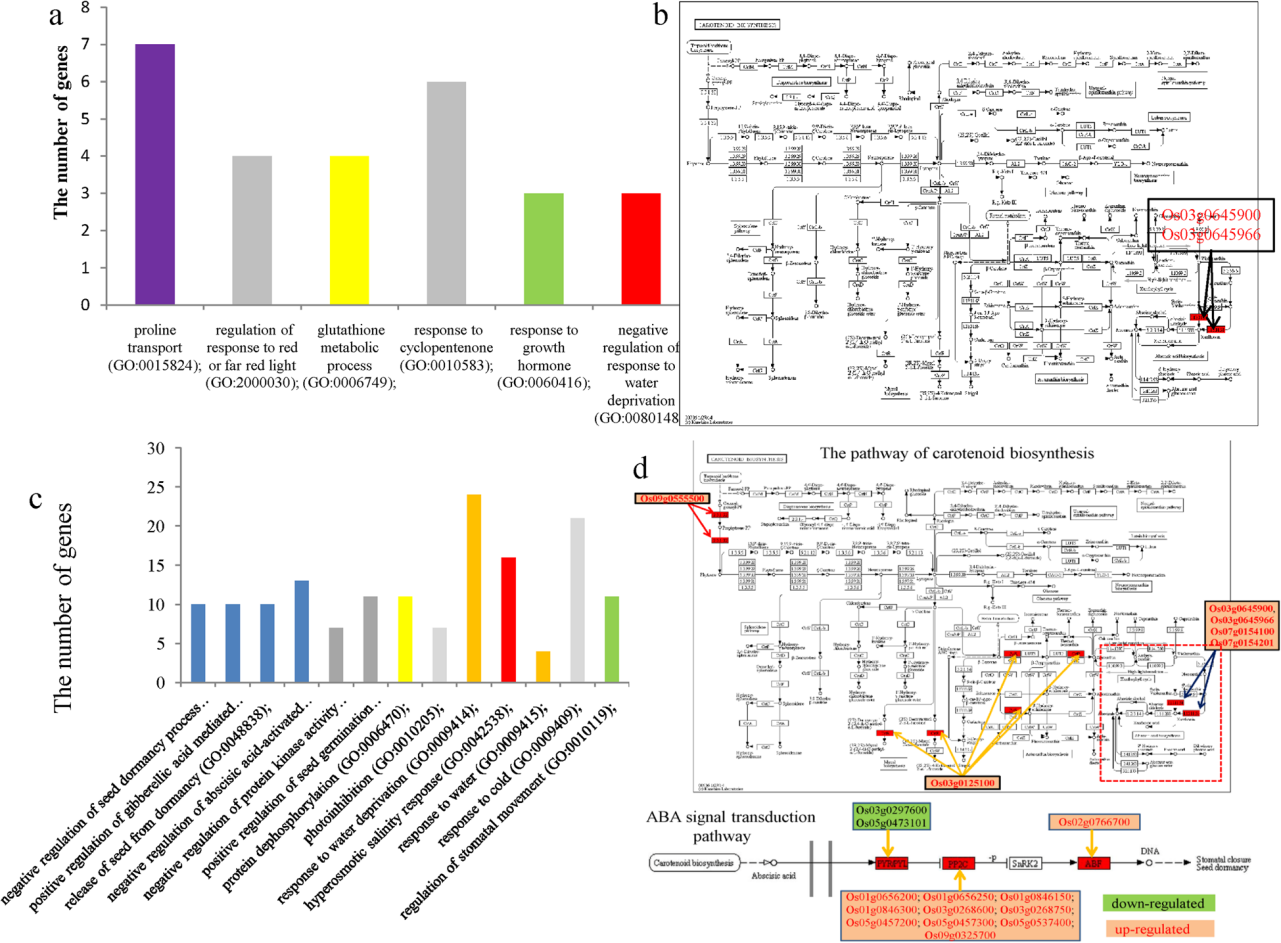
The enrichment analysis of overlapped DEGs between the germination and seedling stages. **a**: Sixty-nine DEGs were enriched in biological processes between the germination and seedling stages; **b**: 69 enriched DEGs overlapped in the metabolic pathways between the germination and seedling stages; **c**: 164 DEGs were enriched in biological processes that were shared among three sampling points at the seedling stage; b: 164 DEGs were enriched in metabolic pathways that were shared among three sampling points at the seedling stage

We also investigated the overlapping DEGs between germination and seedling stages and among the three sampling points at the seedling stage. Some of the 69 shared DEGs between germination and the seedling stage were enriched in six GO terms (FDR < 0.01) (Fig. 5a), such as proline transport (GO:0015824) (seven genes), glutathione metabolic process (GO:0006749) (four genes), and negative regulation of response to water deprivation (GO:0080148) (three genes). The KEGG analysis indicated that only two genes, Os03g0645900 (*OsNCED3*) and Os03g0645966, were significantly enriched in the carotenoid biosynthesis pathway (ko00906) (FDR < 0.01) (Fig. 5b). The 164 overlapping genes among the three sampling points at the seedling stage were clustered into 13 GO terms (FDR < 0.001) (Fig. 5c), including negative regulation of abscisic acid-activated signaling pathway (GO:0009788) (13 genes), response to water deprivation (GO:0009414) (24 genes), hyperosmotic salinity response (GO:0042538) (16 genes), response to water (GO:0009415) (four genes), and regulation of stomatal movement (GO:0010119) (11 genes). These DEGs were also significantly grouped into the pathways of plant hormone signal transduction (ko04075, 13 genes) and carotenoid biosynthesis (ko00906, six genes) (FDR < 0.00001) (Fig. 5d). All of the 13 DEGs enriched for plant hormone signaling pathways are involved in abscisic acid (ABA) signal transduction (Fig. 5d). Of them, two DEGs (Os03g0297600 and Os05g0473101) belongs to the PYR1/PYL family, 10 genes (Os01g0656200, Os01g0656250, Os01g0846150, Os01g0846300, Os03g0268600, Os03g0268750, Os05g0457200, Os05g0457300, Os05g0537400, and Os09g0325700) belong to the protein phosphatase 2C family, and one gene (Os02g0766700) encodes a bZIP transcription factor acting as an ABA responsive element binding factor.

### Expression Patterns of Shared Genes among Three Sampling Points in the Seedling Stage

**Fig. 6:**
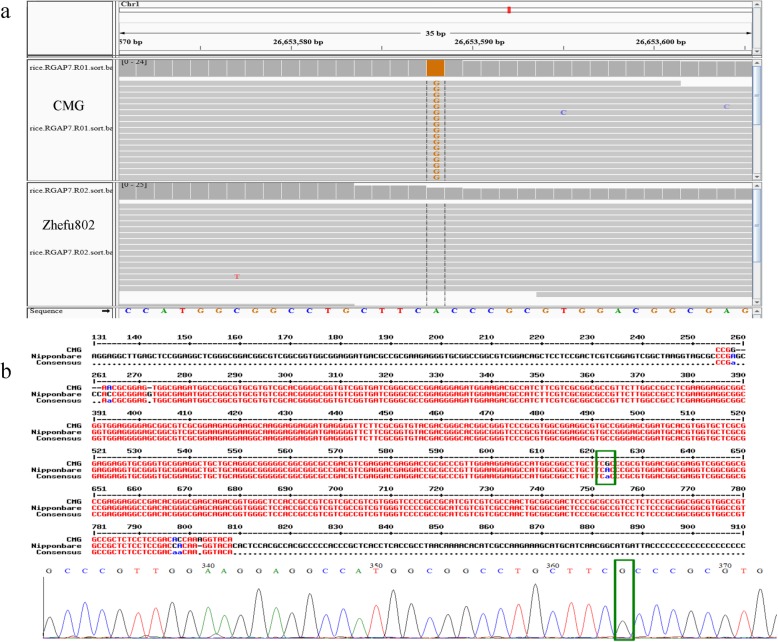
Detection and validation of the candidate gene *OsPP2C* tolerant to salt at the seedling stage. **a**: The detected sequence variant of *OsPP2C* based on genome sequencing; **b**: sequencing and alignment of amplified PCR products using the specific primers for *OsPP2C*

We further analyzed the expression patterns of DEGs among three sampling points at the early seedling stage according to four expression patterns: Up-Up-Up (U-U-U), Down-Down-Down (D-D-D), Down-Up-Down (D-U-D), and Up-Down-Up (U-D-U). The number of DEGs in the expression patterns U-U-U, D-D-D, D-U-D, and U-D-U were 92, 10, 16, and 29, respectively (Fig. 6a,Additional file 1: TableS5). The DEGs in the expression patterns D-U-D and U-D-U were not significantly enriched in any KEGG pathway. Some DEGs in the expression pattern U-U-U were grouped into the two pathways of plant hormone signal transduction (ko04075) (11 genes: Os01g0656200, Os01g0656250, Os01g0846150, Os01g0846300, Os03g0268600, Os03g0268750, Os05g0457200, Os05g0457300, Os05g0537400, Os09g0325700, and Os02g0766700) and carotenoid biosynthesis (ko00906) (six genes: Os09g0555500, Os03g0125100, Os03g0645900, Os03g0645966, Os07g0154100, and Os07g0154201) (Fig. 5d, marked in red). Two DEGs (Os03g0297600 and Os05g0473101) in the expression pattern D-D-D were also clustered into the plant hormone signal transduction pathway (Fig. 5d, marked in green).

We also investigated the transcription factors (TFs) involved in the four expression patterns. We detected 10 TFs including two ABA-inducible bHLH-type TFs (Os01g0705700 and Os01g0705750), two C-2b heat shock TFs (Os06g0553001 and Os06g0553100), one ethylene-responsive TF RAP2–3 (Os05g0361700), one GAMYB TF (Os04g0508500), one MYB23 TF (Os02g0618400), one NAC TF ONAC010 (Os03g0327800), one probable WRKY TF 57 (newGene_197), one HEC2 TF (Os09g0455300), and 1 bZIP TF BZIP23 (Os02g0766700) (Fig. 6d). All of these TFs were significantly up-regulated at three sampling points in the seedling stage.

### Screening the Target Candidate Genes for Salinity Tolerance

In 31 possible candidate genes including 23 genes containing nonsynonymous coding SNPs and eight frameshift coding genes (Table 5), nonsynonymous and frameshift mutations simultaneously occurred in Os01g0712250 and Os01g0655700. One gene (Os01g0655400) encodes a putative transposon protein and 13 genes encode hypothetical proteins. We investigated the expression of the remaining 17 genes using transcriptomic sequencing. Only Os01g0656200 (*OsPP2C8*), encoding a protein phosphatase 2C family protein (PP2C), had significantly increased expression levels at the young seedling stage; the expression levels were not altered at the germination stage under the salt treatment. Compared with normal growth conditions, the expression of other genes did not change in the salt treatment. We also compared the sequence of Os01g0656200 (*OsPP2C8*) in CMG and Zhefu802 according to BSA sequencing, and we detected one SNP (A in Zhefu802 to G in CMG) in the CDS region of *OsPP2C8*causing the alternation of the corresponding amino acid (Thr in Zhefu802 to Ala in CMG) (Fig. 6a). We also developed the corresponding primers for the variant site and validated the variant site by sequencing and aligning PCR products from CMG (Fig. 6b). Hence, *OsPP2C8* was thought to be the most likely candidate gene for conferring salinity tolerance in the seedling stage in CMG.

## Discussion

Rice is considered relatively tolerant to salt at germination and sensitive to salinity at the young seedling and reproductive stages (Heenanet al., 1988; Khatun et al., 1995; Zeng et al., 2001). Rice salinity tolerance is also thought to be a quantitative trait. Conventional QTL analysis is a laborious and time-consuming process because of the requirement of genotyping and phenotyping a large number of individuals derived from a biparental cross (Lim et al., 2014). Whole genomic sequencing assisted BSA has been widely used for the analysis of quantitative traits controlled by a few major genes and is thought to be less useful for the identification of minor QTLs (Guo et al., 2017). In this study, we performed a BSA analysis based on whole genome sequencing and transcriptome sequencing of rice subjected to salt stress to identify the major QTLs for salinity tolerance in the rice landrace CMG, which has a strong tolerance to salinity.

### Mapping Candidate Regions for Salinity Tolerance in CMG

In the whole genome sequencing analysis, we detected a large number of SNPs and InDelInDels between CMG and Zhufu802 and obtained six candidate regions for salinity tolerance in CMG. In these six regions, three regions are small (approximately 20 kb; Chr1: 26110000–26,130,000, 26,160,000–26,180,000, and 36,140,000–36,160,000) and contained two to three putative genes, respectively. Os01g0648000 (located in the region from 26,110,000–26,130,000 on Chr1) encodes a putative potassium channel protein that has been previously shown to be associated with salt stress (Fuchs et al., 2005); however, it was not differentially expressed in our transcriptase analysis.

Another two candidate regions are large (0.81 Mb spanning 26,490,000–27300000and 1.19 Mb from 29,440,000–30,630,000 on Chr1). In the candidate region from 26,490,000–27,300,000, we detected a sequence variant in the CDS region of Os01g0656200, the expression of which was upregulated in the seedling stage under the salt stress treatment. In agreement with these results, a previous study also showed that Os01g0656200 was associated with salt tolerance in different salt tolerant rice lines (Walia et al., 2005; Pandit et al., 2010). Os01g0656200 (*OsPP2C8*) encodes a type of protein phosphatase 2C family protein (PP2C). PP2C was known to be involved in abscisic acid signal transduction in higher plants (Sheen, 1998). Three *PP2Cs* (*AtPP2CG1*, *AtPP2CA*, and *OsPP2C51*) were found to enhance salinity tolerance in an abscisic acid-dependent mannerin Arabidopsis and promoted seed germination in rice (Liu et al., 2012; Cui et al., 2013; Bhatnagar et al., 2017). We detected a high level of ABA in the leaves and roots of CMG under salt stress in the seedling stage and the expression of *OsPP2C8* was significantly up-regulated at the three sampling points in the seedling stage. CMG shows strong salinity tolerance in the germination and seedling stages, and the expression of this gene was significantly up-regulated in the seedling stages under salt stress. Hence, *OsPP2C8* is also considered the most likely candidate gene for salt tolerance in CMG.

### Plant Hormone and Salinity Tolerance

Plant hormones are thought to be the most important endogenous substances for regulating various physiological responses that lead to adaptation to salinity (Pearce et al., 2001b). Yang et al. (2014) assayed the levels of gibberellin, cytokinin, auxin, and abscisic acid under salt stress in tomato, and the study indicated that ABA played a major role in tomato salt tolerance. The exogenous application of ABA was found to offset the effects of osmotic and ion stress from salt stress conditions in commonbean (Khadri et al., 2007), wheat (Gurmani et al., 2013), and rice (Sripinyowanich et al., 2013) by reducing the sodium concentration and improving osmotic adjustment. In this study, we also found that the ABA level largely increased in the leaves and roots, even after approximately 30 min under salinity stress. Overlapping DEGs among three seedling sampling points were enriched in the pathways of carotenoid biosynthesis and ABA signal transduction, and two DEGs shared in the germination and seedling stages were also clustered into carotenoid biosynthesis. Duan et al. (2012) reported that the genes responsive to salt stress in the tomato line ‘Moneymaker’ and its wild genotype PI365967 (tolerant to salt) were involved in the carotenoid and ABA biosynthetic pathway. Similar results were also obtained in a close relative of Arabidopsis, *Thellungiella*, which showed strong tolerance to salt (Wong et al., 2006).

Combined with the above studies, these results likely imply that similar mechanisms in the adaptation to salinity stress apply for both cultivated plants and wild species, namely, rapid accumulation of ABA upon salinity stress condition set off the ABA-dependent signal transduction pathway to activate downstream target genes to respond to salt stress.

The transcriptome analysis under salt stress indicated that only two genes *OsNCED3* (Os03g0645900) and Os03g0645966, a hypothetical gene of 69 DEGs were significantly clustered into the pathway of carotenoid biosynthesis. Interestingly, *OsNCED3* was down-expressed in the germination stage and up-expressed at three sampling points in the seedling stage. Overexpression of rice *OsNCED3* increased the accumulation of ABA, reduced relative water loss, and delayed seed germination in Arabidopsis (Hwang et al., 2010). In the sequence variants of the two genes in the BSA-seq analysis, we found that there were three nonsynonymous SNPs in the CDS regions. It is possible that down-expression of *OsNCED3* decreases the level of ABA to promote seed germination in CMG in the germination stage. In addition, no shared DEGs among the germination and seedling stages were identified in plant hormone signaling pathways, which seems to indicate that the mechanism of salinity tolerance in the germination stage might be different from that in the seedling stage. Hence, *OsNCED3* could be invovled into salinity tolerance in CMG at the germination stage, but it was not mapped into the candidate region based on the mapping population of CMG/Zhefu802.

Among 13 DEGs enriched in the ABA signal transduction pathway in the seedling stage, one (Os02g0766700), two (Os03g0297600 and Os05g0473101), and 10 genes belong to the ABF (ABRE binding factors), PYR/PYL, and PP2C families, respectively. Os02g0766700 encodes a bZIP transcription factor. Previous studies have indicated that bZIP transcription factors play crucial roles in the ABA signaling pathway in plants (Amir Hossain et al., 2010; Liu et al., 2014). The PYR/PYL family has been identified as ABA receptors (Liu et al., 2012; Ludwików, 2015). In the model of ABA signaling in plants, PP2Cs first interact with SnRK2s to form a reversible regulatory module in the manner of ABA-independence (Umezawa et al., 2009). The PYR-PP2C-SnRK2 complex is the primary framework for ABA signaling and phosphorylates downstream substrates, including bZIP transcription factors to activate ABA-responsive gene expression. We detected a sequence variant in the CDS region of *OsPP2C8* from CMG, Zhefu802, and Nipponbare to alter the corresponding amino acid, which could cause the activation of the downstream genes in the pathway of ABA signaling to increase the salinity tolerance of CMG.

## Materials and Methods

### Materials

A rice salinity tolerant landrace, Changmangu (CMG), was collected from a coastal beach in Zhanjiang city, Guangdong Province, China. Zhefu802 is a rice cultivar that is sensitive to salinity.

Approximately 2000 plants in the F_2_ population were derived from a cross between CMG and Zhefu802. Their leaves were harvested for BSA-seq to map the salinity tolerant genes. The seeds from the F_2_ plants (F_2:3_) were also collected to identify salinity tolerance. The seeds of more than 1000 F_2:3_ lines from the F_2_ population and two parents were immersed in 120 mM NaCl solution for 4 days to investigate germination rate at 25 °C in the artificial climate incubator (MGC-450HP). The F_2:3_ lines with more than 40% germination rate continued to be cultivated under salt stress for 21 days. Thirty F_2:3_ lines growing well were selected and their corresponding F_2_ plant leaves were used to construct the salt tolerant bulk.

### Bulked Segregant Analysis by Pooled Sequencing

BSA-seq was used to identify the genes regulating the tolerance to salinity in the above mentioned F_2_ population. We selected 30 extremely tolerant and 30 F_2_ plants sensitive to salinity to create extreme sample pools. Genomic DNA was extracted using a modified CTAB (Hexadecyltrimethylammoniumbromide) method and purified by chloroform: phenol (1:1) (Chen and Ronald 1999). The DNA quality was checked using an Agilent bioanalyzer 1000 (Agilent Technologies, Singapore). Library preparation was performed according to the manufacturer’s protocol. Genomic re-sequencing was conducted to generate paired-end 100-base (PE100) reads using the Illumina Hiseq 2000 platform (Illumina Technologies), which was conducted by Biomarker (China). Clean reads were aligned to reference genome sequences of the *Japonica* rice Nipponbare genome (http://ftp.ensemblgenomes.org/pub/release-24/plants/fasta/oryza_sativa/dna/Oryza_sativa.IRGSP-1.0.24.dna.toplevel.fa.gz) using BWA software (Li and Durbin, 2009). SNPs and small InDelInDels were detected using GATK software (McKenna et al., 2010). The tool of Mark Duplicate in Picard (http://sourceforge.net/projects/picard/) was used to eliminate PCR duplication to increase SNP/InDel-calling accuracy. SNP/InDel-index was calculated for all the SNP/InDel positions. We excluded SNP/InDel positions with multiple genotypes and read depth < 4 from the two bulk sequences. The association analysis was conducted by Euclidean Distance (ED) and SNP/InDel-index, respectively (Hill et al., 2013; Fekih et al., 2013). The overlapped regions based on the above two methods were considered candidate regions for salinity tolerance.

### Sample Preparation and Transcriptome Sequencing

Dry seeds from CMG were immersed in 120 mMNaCl solution and sterile water for 4 days. The young buds and roots were harvested at germination and rapidly stored in liquid nitrogen for transcriptomic sequencing. Thirty-day-old seedlings in nutrient solution were treated with 120 mM NaCl solution for 0 min, 30 min, 3 h, and 24 h; approximately 2 g of roots per treatment were collected and rapidly stored in liquid nitrogen for transcriptomic sequencing of the seedling stage. Total RNA samples were extracted using the TRIzol reagent (Invitrogen) and then treated by RNase-freeDNase I (Takara) to remove genomic DNA. mRNA libraries were created according to the standardprotocols provided by Illumina. The mRNA quality including mRNA concentration and fragment size was tested using Qubit2.0 and Agilent 2100.mRNA was enriched using Dynabeads oligo (dT) (Dynal; Invitrogen) and fragmented using fragmentationbuffer. Double-stranded cDNAs were produced using reversetranscriptase (Superscript II; Invitrogen) and random hexamerprimers and further purified using AMPure XP beads. The purified double-stranded cDNA samples were enriched by PCR to construct the final cDNA libraries for sequencing using Hiseq 2500 (150 bp paired ends) by Biomarker (China). All raw-sequence reads data were uploaded to NCBI SequenceRead Archive (SRA, http://www.ncbi.nlm.nih.gov/Traces/sra) with accession numbers SRP143635.

Clean reads were also aligned toreference genome sequences of the *Japonica* rice Nipponbare genome using TopHat2 (Kim et al., 2013). Gene expression differences among different sampling points were detected using the EBSeq package (v1.10.1). Flod change≥2 and False Discovery Rate (FDR) < 0.01 were set to act as the standard for screening the DEGs. Functional classification of DEGs, including Gene Ontology and KEGG pathways, were analyzed using the GOseq R package (Release2.12) and KOBAS software (v2.0).

### Real-Time PCR Confirmation of DEGs

A total of 30 DEGs were randomly selected to confirm the transcriptomic sequencing results using real time PCR (RT-PCR). The corresponding sequences of these genes were obtained from the rice genome sequence database (Rap-db). These primers were designed according to the CDS sequences of the corresponding genes using Primer3 software (http://frodo.wi.mit.edu/) (Table S1). The *Osactin1* gene was used as the internal control. Total RNAs were isolated from thesame samples for transcriptomic sequencing using the TRIzol reagent (Invitrogen) for RT-PCR. First-strand cDNA was synthesized from 1 mg of DNase I-treated RNA samples in a 20 μl reaction solution with the oligo (dT) primer, using a Rever TraAce-akit (TOYOBO). Standard RT–qPCR was performed using SYBR Green SuperMix (Bio-Rad) on a CFX96 Real Time System (BioRad).

### Assays of abscisic Acid Level

Thirty-day-old seedlings of Zhefu802 and CMG were treated in 120 mM NaCl solution for 0 min (mock), 30 min (SSI), 3 h (SSII), and 24 h (SSIII). Ten young seedlings were prepared at every sampling point to assay the level of ABA. The ABA content was scored using the plant abscisic acidELISA kits (AndyGene) according to the manufacturer’s protocol.

## Conclusions

In this study, we mapped six candidate regions for salt tolerance on chromosome 1 based on BSA-seq using extreme populations and identified 32 candidate genes according to the sequence polymorphism in the regions of promoters and ORFs between CMG and Zhefu802. The transcriptome analysis under the conditions of salt stress and normal growth identified numerous DEGs at the germination and young seedling stages. We investigated the expression of the 32 candidate genes and found that *OsPP2C8* (Os01g0656200) was differentially expressed in the seedling stage under salt stress. Hence, *OsPP2C8* was identified as the target candidate gene for salinity tolerance in the seedling stage, which will provide an important genetic resource for salt tolerant rice breeding.

## Supplementary information

**Additional file 1: Table S1.** The mapped candidate regions based on SNP-index and ED. **Table S2.** The mapped candidate regions based on InDel-index and ED. **Table S3.** The overlapped candidate regions based on polymorphic SNPs and InDels. **Table S4.** Characteristics of the RT-PCR primers for validating the results of transcriptomic sequencing. **Table S5.** Functional annotation of the DEGs in four types of expression patterns at the seedling stage

## Data Availability

All data generated or analysed during this study are included in this published article and its supplementary information files.

## Abbreviations

ABA: Abscisic acid
bHLH: Basic helix-loop-helix
BSA: Bulked segregant analysis
bZIP: Basic leucine zipper
DEG: Differentially expressed gene
ED: Euclidean distance
FDR: False discovery rate
GAMYB: GA-regulated myeloblastosis
NAC: NAM (no apical meristem), ATAF, CUC (cup-shaped cotyledon)
NGS: Next generation sequencing
ORFs: Open reading frames
PP2C: Proteinphosphatase 2C
PYR1/PYL: Pyrabactin resistance 1/PYR1-like
RAP: RNA Polymerase II-associated Protein
SNP: Single nucleotide polymorphism;
TFs: Transcription factors
