# Supplementary material for: Genomic and transcriptomic analysis reveal molecular basis of salinity tolerance in a novel strong salt-tolerant rice landrace Changmaogu
Source: Rice (N Y). 2019 Dec 27;12:99. doi: 10.1186/s12284-019-0360-4 (PMC6934643; doi:10.1186/s12284-019-0360-4)
Supplement: Supplementary file 1 — Additional file 1: Table S1. The mapped candidate regions based on SNP-index and ED. Table S2. The mapped candidate regions based on InDel-index and ED. Table S3. The overlapped candidate regions based on polymorphic SNPs and InDels. Table S4. Characteristics of the RT-PCR primers for validating the results of transcriptomic sequencing. Table S5. Functional annotation of the DEGs in four types of expression patterns at the seedling stage [file 12284_2019_360_MOESM1_ESM.docx]

Table S1 The mapping candidate regions based on SNP-index and ED

| Variant Type | Chromosome ID | Start | End | Size (Mb) |
| --- | --- | --- | --- | --- |
| ED | Chr1 | 23320000 | 27390000 | 4.07 |
|  | Chr1 | 29340000 | 30980000 | 1.64 |
|  | Chr1 | 32360000 | 32600000 | 0.24 |
|  | Chr1 | 32840000 | 33090000 | 0.25 |
|  | Chr1 | 34790000 | 37640000 | 2.85 |
|  | Chr1 | 37670000 | 37680000 | 0.01 |
|  | Chr12 | 26400000 | 27500000 | 1.1 |
|  | Total |  |  | 10.16 |
| Index | Chr1 | 24650000 | 24730000 | 0.08 |
|  | Chr1 | 25910000 | 27310000 | 1.4 |
|  | Chr1 | 29440000 | 30630000 | 1.19 |
|  | Chr1 | 35770000 | 35940000 | 0.17 |
|  | Chr1 | 36140000 | 36160000 | 0.02 |
|  | Chr1 | 36580000 | 37380000 | 0.8 |
|  | Total |  |  | 3.66 |

Table S2 The mapping candidate regions based on InDel-index and ED

| Variant Type | Chromosome ID | Start | End | Size (Mb) |
| --- | --- | --- | --- | --- |
| ED | Chr1 | 15290000 | 15660000 | 0.37 |
|  | Chr1 | 22270000 | 22600000 | 0.33 |
|  | Chr1 | 22620000 | 22630000 | 0.01 |
|  | Chr1 | 22660000 | 27370000 | 4.71 |
|  | Chr1 | 29330000 | 30950000 | 1.62 |
|  | Chr1 | 32380000 | 32530000 | 0.15 |
|  | Chr1 | 32860000 | 33070000 | 0.21 |
|  | Chr1 | 35040000 | 36260000 | 1.22 |
|  | Chr12 | 26560000 | 27520000 | 0.96 |
|  | Total |  |  | 9.58 |
| Index | Chr1 | 26110000 | 26130000 | 0.02 |
|  | Chr1 | 26160000 | 26180000 | 0.02 |
|  | Chr1 | 26490000 | 27300000 | 0.81 |
|  | Chr1 | 29390000 | 30800000 | 1.41 |
|  | Chr1 | 35800000 | 35960000 | 0.16 |
|  | Chr1 | 35980000 | 36160000 | 0.18 |
|  | Chr1 | 36500000 | 36520000 | 0.02 |
|  | Chr1 | 36540000 | 37510000 | 0.97 |
|  | Chr1 | 37570000 | 37570000 | 0 |
|  | Chr1 | 37590000 | 37630000 | 0.04 |
|  | Total |  |  | 3.63 |

Table S3 The overlapped candidate regions based on polymorphic SNPs and InDels, respectively

| Variant Type | Chromosome ID | Start | End | Size (Mb) |
| --- | --- | --- | --- | --- |
| SNP | Chr.1 | 24650000 | 24730000 | 0.08 |
|  | Chr.1 | 25910000 | 27310000 | 1.4 |
|  | Chr.1 | 29440000 | 30630000 | 1.19 |
|  | Chr.1 | 35770000 | 35940000 | 0.17 |
|  | Chr.1 | 36140000 | 36160000 | 0.02 |
|  | Chr.1 | 36580000 | 37380000 | 0.8 |
|  | Total |  |  | 3.66 |
| InDel | Chr.1 | 26110000 | 26130000 | 0.02 |
|  | Chr.1 | 26160000 | 26180000 | 0.02 |
|  | Chr.1 | 26490000 | 27300000 | 0.81 |
|  | Chr.1 | 29390000 | 30800000 | 1.41 |
|  | Chr.1 | 35800000 | 35960000 | 0.16 |
|  | Chr.1 | 35980000 | 36160000 | 0.18 |
|  | Total |  |  | 2.6 |

Table S4 The character of the RT-PCR primers for validating the results of transcriptomic sequencing

| Gene ID | Forward primers (5'to 3') | Reverse primes (5'to 3') | Product size (bp) |
| --- | --- | --- | --- |
| Os01g0135700 | TGTCGAACACCACCGAGAAG | GAAGACCCTCTCGATCTCCG | 95 |
| Os01g0656200 | TCCTCGGTGTCCTTGCTACT | GAGGAACTCGTCCTTGTCCG | 113 |
| Os01g0699400 | CCAAGTGCTCAACGAGTCCA | CCCTCGGGATCAACACACTC | 144 |
| Os01g0705700 | TGGCGTCCATGTACTTCTCC | GAGAGACGAGCGAACGTACC | 143 |
| Os01g0756300 | ACCGTACCTCTCCGAGTTCT | CATGCCGTCCATGTACCACT | 106 |
| Os01g0846300 | ATGGATGGGGAGGTCGGTAA | GACCTGTGTTGGGCTAACGA | 138 |
| Os02g0179600 | GAACTGGGGCATGAACACCA | CCTGGTCGAACTCCATGTCG | 131 |
| Os02g0618400 | TTGTGGGGAAACAGGTGGTC | CTCGATGTTCTTGGACGGCT | 117 |
| Os02g0682300 | CCCTCTGGTGTGCTACTGC | ATCCCGACGACGAAGAGCAT | 108 |
| Os02g0766700 | ATGTGTTCCCTCCGATGGTG | TTCCATCTTGCCGAAGCCAT | 140 |
| Os03g0197100 | CATCTACTCGCTGTTCGGCT | ATGAGGAACGCGTAGTTCGG | 141 |
| Os03g0327800 | AGCGCAAGAGATCATCGGAC | AGTTGCACCAACGCATGAAC | 101 |
| Os04g0508500 | AGCCGTCCAAGAACATCGAG | TCTCGGCAACGATGATGTCC | 138 |
| Os04g0585050 | GGACCTCGCTGTAGTGTGTC | GATCCTCGTCCTCCAGCTTC | 110 |
| Os05g0361700 | ATGTGTGGGGGAGCGATCAT | GAACGACTCGTCACCGGC | 120 |
| Os05g0381400 | TTGATCGTGATCGGGTTCGC | ACGAGGAAGTAGAAGGTGGC | 104 |
| Os05g0457200 | TCCATCAGGCCGGACTTCCT | TGCAATCTGTTTCTTGCGTGC | 120 |
| Os06g0553100 | GTGTGCGTGTGTTTTGGGAG | TGGGTCCCAAACTCTGTTGT | 127 |
| Os09g0332300 | GGATTGCTTGGCTTGAGTGG | CATGACAATGGCTGCTGCTC | 141 |
| Os09g0455300 | AGCTTGACCTGATGAGCACG | AGAACTGGGGAGAAGGGGTC | 126 |
| Os01g0908600 | GGAAGAGCACTCAAGGCGAT | TCCTCATGGCGGAAATGGTC | 136 |
| Os03g0297600 | TGGTGGAGTCCTACGTCGTC | GTCTTGGCGAGAGACTGGAG | 106 |
| Os10g0552800 | ATCGACGCCTCACTACGGC | CGACCTTGGCCTTGACGAG | 97 |
| Os01g0655500 | CGCCGACAGATGATGTACCA | ACCAGTGGCGCAAAGAGTAA | 136 |

Table S5 The functional annotation of the DEGs in four types of expression patterns at the seedling stage

|  | Gene ID | FDR | | | Functional annotation |
| --- | --- | --- | --- | --- | --- |
|  |  | SSI | SSII | SSIII |  |
| U-U-U | Os01g0135700 | 2.36E-09 | 2.00E-13 | 2.43E-08 | Probable calcium-binding protein CML16 |
|  | Os01g0206300 | 9.43E-07 | 4.33E-11 | 5.45E-05 | Putative CBL-interacting protein kinase 13 |
|  | Os01g0214500 | 0 | 3.10E-09 | 2.60E-10 | Conserved hypothetical protein |
|  | Os01g0556650 | 0.00207636 | 0.00257709 | 2.54E-11 | Nitrate transporter 1.4 |
|  | Os01g0644200 | 1.86E-08 | 0 | 5.86E-10 | Similar to Little protein 1 |
|  | Os01g0656200 | 2.38E-09 | 0 | 2.80E-14 | Probable protein phosphatase 2C |
|  | Os01g0656250 | 0.00186229 | 0 | 2.72E-06 | Probable protein phosphatase 2C |
|  | Os01g0699400 | 0 | 0 | 0 | Mitogen-activated protein kinase kinase kinase NPK1 |
|  | Os01g0699500 | 0 | 1.13E-09 | 4.95E-08 | Mitogen-activated protein kinase kinase kinase NPK1 |
|  | Os01g0705200 | 3.23E-13 | 0 | 0 | Late embryogenesis abundant protein, group 3 |
|  | Os01g0705700 | 0.00084878 | 1.31E-06 | 0 | Transcription factor ABA-INDUCIBLE bHLH-TYPE |
|  | Os01g0705750 | 0 | 1.72E-12 | 0 | Transcription factor ABA-INDUCIBLE bHLH-TYPE |
|  | Os01g0756300 | 0.00011292 | 0 | 2.58E-09 | Arginine/serine-rich protein 45 |
|  | Os01g0802600 | 4.36E-09 | 0 | 1.55E-15 | EID1-like F-box protein 3 |
|  | Os01g0832600 | 0 | 0 | 0 | Flavonol synthase/flavanone 3-hydroxylase |
|  | Os01g0846150 | 0 | 5.55E-16 | 6.74E-14 | Probable protein phosphatase 2C |
|  | Os01g0846300 | 0 | 1.11E-15 | 5.75E-12 | Probable protein phosphatase 2C |
|  | Os01g0854000 | 1.24E-07 | 0 | 9.99E-16 | Uncharacterized acetyltransferase At3g50280 |
|  | Os01g0931600 | 9.48E-05 | 0.00385269 | 0.00459219 | Protein LURP-one-related 11 |
|  | Os01g0954000 | 4.21E-12 | 1.35E-05 | 1.38E-06 | Probable NADPH:quinone oxidoreductase 2 |
|  | Os02g0179600 | 0.00019408 | 2.55E-06 | 0 | Proline-rich receptor-like protein kinase PERK10 |
|  | Os02g0618400 | 0 | 0.00033363 | 0 | Transcription factor MYB23 |
|  | Os02g0618525 | 0 | 0.00273052 | 3.86E-06 | Hypothetical protein |
|  | Os02g0638650 | 6.68E-14 | 0.0022766 | 2.08E-11 | Dynein assembly factor 3 |
|  | Os02g0649300 | 0 | 0 | 0 | Homeobox-leucine zipper protein HOX24 |
|  | Os02g0682300 | 0 | 0.00724756 | 1.54E-10 | E3 ubiquitin-protein ligase RHA1B |
|  | Os02g0686100 | 0 | 0.00108682 | 0 | RING-H2 finger protein ATL80 |
|  | Os02g0719600 | 0 | 0 | 0 | Salicylate O-methyltransferase |
|  | Os02g0766700 | 1.78E-09 | 0 | 0 | ABSCISIC ACID-INSENSITIVE 5-like protein 5 |
|  | Os02g0828200 | 0.00114413 | 0.00016725 | 4.17E-08 | Leucine-rich repeat extensin-like protein 5 (Precursor) |
|  | Os03g0125100 | 3.03E-12 | 2.15E-11 | 1.44E-15 | Beta-carotene 3-hydroxylase, chloroplastic (Precursor) |
|  | Os03g0141200 | 5.32E-08 | 0 | 0 | Beta-amylase 1, chloroplastic (Precursor) |
|  | Os03g0183500 | 0 | 0.0056674 | 0 | Hypothetical protein |
|  | Os03g0197100 | 0.00050035 | 1.49E-12 | 2.81E-08 | Polyol transporter 5 |
|  | Os03g0197125 | 3.28E-07 | 0 | 1.14E-14 | Polyol transporter 5 |
|  | Os03g0268600 | 0 | 0 | 0 | Probable protein phosphatase 2C |
|  | Os03g0268750 | 0 | 0 | 0 | Probable protein phosphatase 2C |
|  | Os03g0289800 | 2.63E-10 | 3.92E-09 | 9.88E-15 | Leucoanthocyanidin dioxygenase |
|  | Os03g0289850 | 2.44E-10 | 2.23E-09 | 0 | Probable 2-oxoglutarate/Fe(II)-dependent dioxygenase |
|  | Os03g0296200 | 0.00016154 | 0 | 0 | Extensin (Precursor) |
|  | Os03g0305600 | 0.0090577 | 2.34E-05 | 0 | Outer envelope pore protein 16-2 |
|  | Os03g0327800 | 0 | 6.21E-08 | 6.77E-15 | NAC transcription factor ONAC010 |
|  | Os03g0341300 | 0 | 0 | 0 | Bidirectional sugar transporter SWEET16 |
|  | Os03g0645900 | 3.33E-16 | 0 | 2.88E-07 | 9-cis-epoxycarotenoid dioxygenase 1 |
|  | Os03g0645966 | 5.57E-11 | 1.76E-13 | 0.00017318 | 9-cis-epoxycarotenoid dioxygenase 1 |
| U-U-U | Os03g0718800 | 0 | 2.28E-05 | 0 | Cortical cell-delineating protein (Precursor) |
|  | Os03g0723400 | 8.29E-09 | 0 | 0 | Formin-like protein 16 (Precursor) |
|  | Os03g0820300 | 0 | 1.85E-10 | 0 | Zinc finger protein ZAT12 |
|  | Os03g0820500 | 0 | 0 | 0 | Actin-depolymerizing factor 3 |
|  | Os03g0860100 | 0 | 0.00929324 | 3.22E-07 | Lysine-rich arabinogalactan protein 19 (Precursor) |
|  | Os04g0244800 | 0 | 0 | 0 | Heavy metal-associated isoprenylated plant protein 26 |
|  | Os04g0508500 | 2.11E-15 | 6.05E-10 | 0 | Transcription factor GAMYB |
|  | Os04g0541700 | 5.55E-16 | 0 | 0 | Homeobox-leucine zipper protein HOX22 |
|  | Os04g0585050 | 0 | 0.00790839 | 8.65E-08 | RING-H2 finger protein ATL44 |
|  | Os05g0206000 | 1.68E-10 | 1.82E-12 | 0.00306191 | Hypothetical protein |
|  | Os05g0361700 | 1.33E-06 | 0 | 0 | Ethylene-responsive transcription factor RAP2-3 |
|  | Os05g0381400 | 0 | 0 | 0 | Proline-rich receptor-like protein kinase PERK8 |
|  | Os05g0457200 | 0.00458127 | 0 | 0 | Probable protein phosphatase 2C |
|  | Os05g0457300 | 0.00132767 | 0 | 0 | Probable protein phosphatase 2C |
|  | Os05g0494600 | 5.04E-10 | 2.22E-16 | 3.93E-06 | EID1-like F-box protein 3 |
|  | Os05g0516700 | 5.25E-12 | 1.17E-08 | 1.94E-06 | Arginine/serine-rich protein 45 |
|  | Os05g0537400 | 3.19E-07 | 6.61E-12 | 9.18E-08 | Probable protein phosphatase 2C |
|  | Os05g0541000 | 0 | 5.78E-05 | 2.94E-13 | Hypothetical protein |
|  | Os05g0542500 | 0 | 0 | 0 | Late embryogenesis abundant protein, group 3 |
|  | Os06g0246500 | 3.65E-07 | 0 | 0 | Pyruvate dehydrogenase E1 component subunit alpha-2 |
|  | Os06g0553001 | 2.75E-12 | 1.53E-09 | 0 | Heat stress transcription factor C-2b |
|  | Os06g0553100 | 7.77E-16 | 6.15E-11 | 1.20E-13 | Heat stress transcription factor C-2b |
|  | Os06g0651200 | 2.75E-07 | 0 | 9.90E-11 | Sulfated surface glycoprotein 185 (Precursor) |
|  | Os06g0698300 | 0.00643512 | 5.50E-14 | 0 | Probable protein phosphatase 2C 59 (Precursor) |
|  | Os07g0154100 | 0 | 1.02E-07 | 0 | 9-cis-epoxycarotenoid dioxygenase 1, chloroplastic |
|  | Os07g0154201 | 0 | 0 | 0 | 9-cis-epoxycarotenoid dioxygenase 1, chloroplastic |
|  | Os07g0418600 | 7.00E-12 | 0.00389344 | 0 | Glycine-rich cell wall structural protein (Precursor) |
|  | Os07g0525900 | 0 | 9.09E-11 | 0 | Bisdemethoxycurcumin synthase |
|  | Os07g0602900 | 3.28E-05 | 2.25E-08 | 1.17E-10 | Ninja-family protein |
|  | Os08g0205800 | 0 | 0 | 5.34E-11 | Hypothetical protein |
|  | Os09g0325700 | 0 | 0 | 0 | Probable protein phosphatase 2C |
|  | Os09g0332300 | 1.71E-11 | 0.00152285 | 3.13E-09 | Pleiotropic drug resistance protein 4 |
|  | Os09g0379500 | 1.99E-07 | 0.0015778 | 7.31E-06 | Hypothetical protein |
|  | Os09g0379600 | 4.34E-05 | 0.0004197 | 3.96E-07 | Homeobox-leucine zipper protein HOX25 |
|  | Os09g0445600 | 5.11E-13 | 1.18E-08 | 0.00929532 | oxidoreductase/ transition metal ion binding protein |
|  | Os09g0455300 | 4.32E-06 | 0 | 4.44E-16 | Transcription factor HEC2 |
|  | Os09g0555500 | 0 | 0 | 0 | Phytoene synthase, chloroplastic (Precursor) |
|  | Os10g0492900 | 1.22E-15 | 0 | 0 | Alpha-galactosidase (Precursor) |
|  | Os10g0505900 | 0.00470498 | 0 | 0 | Arginine/serine-rich protein 45 |
|  | Os10g0521000 | 1.68E-09 | 8.03E-11 | 0 | Probable trehalase |
|  | Os10g0542750 | 1.35E-10 | 4.33E-11 | 2.22E-16 | Sulfated surface glycoprotein 185 (Precursor) |
|  | Os10g0548100 | 9.33E-15 | 0 | 0 | Arginine/serine-rich protein 45 |
|  | Os11g0451700 | 0 | 0 | 0 | Embryogenic cell protein 40 |
|  | Os11g0453900 | 0 | 1.11E-12 | 0 | Dehydrin Rab16D |
|  | Os11g0454000 | 6.69E-11 | 0 | 0 | Dehydrin Rab16C |
|  | Os11g0454200 | 1.74E-05 | 0 | 0 | Dehydrin Rab16B |
|  | Os12g0147200 | 9.48E-05 | 0 | 0 | Chitin-binding lectin 1 (Precursor) |
|  | Os12g0168100 | 0 | 1.11E-16 | 4.45E-13 | Formin-like protein 8 (Precursor) |
|  | Os12g0226400 | 1.88E-05 | 3.89E-05 | 1.48E-07 | 2-alkenal reductase (NADP(+)-dependent) |
|  | Os12g0242500 | 0 | 0 | 0 | Vegetative cell wall protein gp1 (Precursor) |
|  | NewGene_197 | 1.09E-07 | 8.53E-05 | 2.95E-09 | Probable WRKY transcription factor 57 |
|  | Os01g0347600 | 9.6428E-11 | 0.00020608 | 6.1952E-10 | Fruit bromelain (Precursor) |
|  | Os01g0908600 | 3.1511E-05 | 2.8915E-12 | 4.0174E-08 | Proline transporter 1 |
|  | Os03g0297600 | 0 | 3.5409E-11 | 4.2983E-11 | Abscisic acid receptor PYL4 |
|  | Os04g0627300 | 2.3364E-07 | 0.00389581 | 0.00943607 | Hypothetical protein |
| D-D-D | Os04g0656800 | 1.8421E-05 | 3.8858E-14 | 2.7692E-05 | Peroxidase 16 (Precursor) |
|  | Os05g0473101 | 1.4369E-08 | 4.5067E-09 | 8.9832E-05 | Abscisic acid receptor PYL4 |
|  | Os05g0548900 | 0 | 9.5525E-09 | 1.0015E-12 | Phosphoethanolamine N-methyltransferase 1 |
|  | Os06g0107800 | 3.6645E-07 | 1.4052E-11 | 0.00128066 | B3 domain-containing protein |
|  | Os10g0552800 | 0.00177785 | 9.2665E-07 | 0 | Cortical cell-delineating protein |
|  | NewGene_123 | 2.2803E-06 | 0.00033838 | 1.6151E-11 | Hypothetical protein |
|  | Os01g0121500 | 7.3695E-07 | 0.0005798 | 0.00114346 | CASP-like protein Os02g0134500 |
|  | Os01g0510200 | 5.5511E-16 | 0.00039274 | 0 | Hypothetical protein |
|  | Os01g0636400 | 1.5987E-13 | 0.00051403 | 3.4568E-06 | Arginine/serine-rich protein 45 |
|  | Os01g0695800 | 0 | 2.2109E-08 | 3.6637E-15 | ABC transporter B family member 9 |
|  | Os01g0879200 | 0 | 4.3215E-05 | 1.5549E-06 | Dirigent protein 11 (Precursor) |
|  | Os02g0770800 | 0 | 0.00106436 | 4.228E-08 | Nitrate reductase [NAD(P)H] |
|  | Os03g0663600 | 1.1698E-11 | 1.602E-09 | 2.8477E-06 | Zeamatin (Precursor) |
|  | Os03g0693800 | 0 | 1.1102E-16 | 3.4247E-09 | Putative germin-like protein 3-4 |
|  | Os03g0793800 | 8.4867E-09 | 8.6739E-05 | 0.00075548 | Non-specific lipid-transfer |
|  |  |  |  |  | protein-like protein |
|  | Os04g0581100 | 9.1223E-05 | 0.00051741 | 0 | Naringenin,2-oxoglutarate 3-dioxygenase |
|  | Os04g0615200 | 0 | 8.4698E-11 | 3.1737E-08 | CYSTM1 family protein A |
|  | Os06g0274800 | 2.6215E-06 | 1.9223E-05 | 0.00068745 | Peroxidase 11 (Precursor) |
|  | Os06g0569500 | 3.3884E-13 | 2.3731E-06 | 4.8406E-06 | Ent-kaurene oxidase, chloroplastic |
|  | Os08g0140300 | 3.2805E-09 | 0 | 2.3519E-06 | Aromatic-L-amino-acid decarboxylase |
|  | Os08g0331800 | 1.0967E-08 | 0 | 0.00772795 | Hypothetical protein |
|  | Os08g0448050 | 5.2461E-06 | 1.1502E-07 | 0.00430789 | Probable 4-coumarate--CoA ligase 5 |
|  | Os08g0508000 | 0 | 1.7847E-07 | 1.1558E-05 | Ent-cassadiene C11-alpha-hydroxylase 2 |
| U-D-U | Os08g0518800 | 0 | 2.2133E-06 | 2.8555E-06 | Xylanase inhibitor protein 2 (Precursor) |
|  | Os09g0255400 | 0 | 8.2104E-06 | 1.9439E-05 | Indole-3-glycerol phosphate synthase |
|  | Os09g0367700 | 0 | 0 | 2.4747E-13 | Probable glutathione S-transferase GSTU1 |
|  | Os10g0113050 | 0 | 1.31E-06 | 0.0001409 | Probable NAD(P)H-dependent oxidoreductase1 |
|  | Os10g0527400 | 0 | 0 | 1.4992E-08 | Probable glutathione S-transferase GSTU6 |
|  | Os10g0528300 | 0 | 0 | 0.00010716 | Probable glutathione S-transferase GSTU6 |
|  | Os10g0528400 | 0 | 0.00037751 | 0.00043596 | Probable glutathione S-transferase GSTU6 |
|  | Os12g0150700 | 0 | 9.0289E-11 | 4.9706E-07 | Purple acid phosphatase 21 (Precursor) |
|  | Os12g0268000 | 2.6312E-14 | 2.8894E-07 | 1.3531E-06 | Cytochrome P450 |
|  | Os12g0268100 | 2.2204E-16 | 2.2001E-08 | 1.6162E-05 | Cytochrome P450 |
|  | Os12g0555200 | 6.6058E-14 | 1.0421E-07 | 1.3989E-14 | Pathogenesis-related protein B |
|  | NewGene_520 | 8.7014E-08 | 0.0089762 | 5.6788E-13 | Hypothetical protein |
|  | Os01g0355250 | 6.0507E-14 | 0 | 0 | Salt stress-induced protein |
|  | Os01g0550800 | 1.1102E-16 | 1.3775E-07 | 1.5735E-08 | -- |
|  | Os02g0582900 | 0 | 7.7309E-12 | 4.5768E-12 | -- |
|  | Os04g0308600 | 1.2505E-05 | 0.00096023 | 0 | Sulfated surface glycoprotein 185 |
|  | Os04g0309900 | 0.00052708 | 0.00835721 | 0 | Sulfated surface glycoprotein 185 |
|  | Os05g0200340 | 2.6534E-07 | 6.0343E-08 | 0.00026001 | 3-epi-6-deoxocathasterone 23-monooxygenase |
|  | Os05g0552550 | 0.00157932 | 4.7334E-10 | 0 | Putative membrane protein ycf1 |
| D-U-D | Os05g0552600 | 4.8806E-05 | 0 | 0 | Pollen-specific leucine-rich repeat |
|  |  |  |  |  | extensin-like protein 3 (Precursor) |
|  | Os06g0169001 | 0 | 2.931E-13 | 9.2371E-14 | Protein GOS9 |
|  | Os06g0352200 | 0.00159605 | 1.5556E-06 | 3.1699E-08 | -- |
|  | Os06g0513050 | 4.1513E-06 | 1.4364E-08 | 0 | -- |
|  | Os07g0104500 | 0 | 0 | 1.2644E-09 | Peroxidase 1 (Precursor) |
|  | Os07g0648000 | 0.00220757 | 0.00089466 | 9.7205E-10 | Formin-like protein 4 (Precursor) |
|  | Os09g0564200 | 0 | 6.347E-05 | 0 | Stem bromelain |
|  | Os10g0122400 | 0 | 2.3152E-07 | 0 | Benzyl alcohol O-benzoyltransferase |
|  | Os10g0323500 | 1.1613E-12 | 2.9571E-06 | 0 | Beta-glucosidase 34 (Precursor) |
